# Effectiveness of Fresh to You, a Discount Fresh Fruit and Vegetable Market in Low-Income Neighborhoods, on Children’s Fruit and Vegetable Consumption, Rhode Island, 2010–2011

**DOI:** 10.5888/pcd12.140583

**Published:** 2015-10-15

**Authors:** Gemma Gorham, Akilah Dulin-Keita, Patricia Markham Risica, Jennifer Mello, George Papandonatos, Amy Nunn, Sara Gorham, Mya Roberson, Kim M. Gans

**Affiliations:** Author Affiliations: Gemma Gorham, Institute for Community Health Promotion, Brown University School of Public Health and Rhode Island Public Health Institute, Providence, Rhode Island; Akilah Dulin-Keita, Patricia Markham Risica, Jennifer Mello, George Papandonatos, Center for Statistical Sciences, Brown University School of Public Health, Providence, Rhode Island; Amy Nunn, Sara Gorham, Mya Roberson, Institute for Community Health Promotion, Brown University School of Public Health, Providence, Rhode Island. Dr Gans is also affiliated with the Rhode Island Public Health Institute, Providence, Rhode Island, and the University of Connecticut, Department of Human Development and Family Studies, and the Center for Health Interventions and Prevention, Storrs, Connecticut.

## Abstract

**Introduction:**

Eating fruits and vegetables is associated with lowered risk for many chronic diseases. However, most Americans, especially members of low-income and minority populations, do not eat adequate amounts. Fresh to You is a public–private partnership program that brings discount fresh produce markets into low-income neighborhoods. We conducted a mixed-methods evaluation of Fresh to You to assess the effect of the program on children’s consumption of fruits and vegetables.

**Methods:**

A local produce distributor brought the Fresh to You markets to 6 community organizations serving low-income families in Rhode Island. The markets, held weekly for 5 months at each site, sold fresh produce at below-retail prices. Parents (N = 480) of children aged 3 to 13 years were recruited at the markets to participate in a 5-month cohort study. The primary outcome was change in children’s fruit and vegetable intake, measured by a validated screener. We also conducted postintervention focus groups at each site with parents and qualitative interviews with site contacts to collect feedback about Fresh to You.

**Results:**

From baseline to 5 months, there was a significant increase in children’s daily fruit and vegetable consumption of 0.48 cups (*t* = 4.16, *P* < .001). Data from follow-up parent surveys, focus groups, and site contact interviews provided positive feedback about Fresh to You and recommendations for improvement.

**Conclusion:**

Fresh to You was effective at increasing consumption of fruits and vegetables among racially and ethnically diverse low-income children aged 3 to 13 years whose parents shopped at the markets. The intervention could serve as a model program for replication in other cities. Refinements and a more rigorous evaluation are needed.

## Introduction

Eating recommended amounts of fruits and vegetables is associated with a lower risk for many chronic diseases (1). However, most Americans, especially low-income Americans and people from racial/ethnic minorities, fall short of eating recommended amounts (2–5). These disparities in fruit and vegetable consumption are partly attributable to the food environment in low-income neighborhoods, where residents often have limited access to affordable, healthful food (6,7).

The Centers for Disease Control and Prevention and the US Department of Agriculture (USDA) recommend increasing access to farmers markets in underserved neighborhoods to address this problem (8,9). However, because many US regions, such as New England, have short growing seasons and because most farmers markets do not operate year-round, this approach has challenges. Furthermore, local farmers may not offer popular fruits or ethnic produce (eg, yucca, plantains), many farmers markets are held in locations that are inaccessible to low-income consumers, and prices at farmers markets are often too high for low-income residents (10). In Rhode Island, only 27.9% of farmers markets accept Supplemental Nutritional Assistance Program (SNAP) payments, and only 39.3% accept vouchers for the Special Supplemental Nutrition Program for Women, Infants, and Children (WIC) Farmers Market Nutrition Program (2). Although some studies have shown that WIC vouchers can increase fruit and vegetable intake (11,12), redemption rates are low, averaging 38.5% in Rhode Island and 55.0% nationally (personal communication, K. Ringleheim, Farm Fresh, RI, May 20, 2014) (13). Financial incentives increase purchases of fruits and vegetables and increase use of SNAP benefits at participating farmers markets (13). However, these strategies do not address issues of year-round produce access or access to culturally appropriate or desirable fruits and vegetables not grown in many US regions.

The Fresh to You program offers a potential solution. The program is a public–private partnership between Brown University and a local distributor of fruits and vegetables. The distributor brought fruit-and-vegetable markets year-round to convenient community locations in low-income neighborhoods, sold the produce (both locally grown and nonlocally grown) to residents at below-retail prices and accepted SNAP benefits. Brown University conducted a mixed-methods (qualitative and quantitative) cohort study of the Fresh to You program to evaluate its effectiveness in increasing fruit and vegetable intake among low-income, ethnically diverse children aged 3 to 13 years whose parents shopped at the markets. The purpose of this study was to assess the effect of the Fresh to You program on the amount of fruits and vegetables children ate.

## Methods

### Study design, target population, and setting

Six community organizations that served low-income Rhode Island families were recruited to operate as Fresh to You markets at 6 sites: 3 elementary schools, a job training site in Providence, a middle school in Central Falls, and a community health center in Woonsocket. All 6 sites were located in low-income census tracts; 3 were located in low-access census tracts in which a significant number of residents lived more than 0.5 miles from the nearest supermarket, and 2 of the latter were located in census tracts defined as having low vehicle access (ie, a significant number of residents did not own motor vehicles) (14) (Table 1).

**Table 1:** US Department of Agriculture Economic Research Service Food Atlas Indicators^a^ Applied to Fresh to You Market Sites in Low-Income Urban Neighborhoods, Rhode Island, 2010–2011

Market Location	Low Income and Low Food Access, Yes/No		Vehicle Availability and Supermarket Access: Low-Income, Low-Access, and Low Vehicle Access, Yes/No
	Low-Income	Low-Income and Low Access (>0.5Mile From Nearest Supermarket)	
Elementary school 1, Providence	Yes	No	No
Elementary school 2, Providence	Yes	Yes	Yes
Elementary school 3, Providence	Yes	Yes	No
Neighborhood health center, Woonsocket	Yes	No	No
Middle school, Central Falls	Yes	No	No
Job training center, Providence	Yes	Yes	Yes

a US Department of Agriculture Economic Research Service (14).

The target population was low-income, racially/ethnically diverse children aged 3 to 13 years. The evaluation used a mixed-methods approach consisting of postintervention focus groups conducted with parents in English and Spanish, postintervention interviews with community organization representatives, collection of sales data at the markets, and preintervention and postintervention surveys with a cohort of parents who shopped at the markets. The study was approved by Brown University’s institutional review board.

### Fresh to You market intervention

The local produce distributor brought produce markets weekly for 5 months to each of the 6 community organizations. Each market lasted 2 hours and offered 23 different produce items, which were set up on tables indoors or outdoors. To ensure quality, fresh produce was purchased daily either from local farmers or from a Rhode Island produce distributor who received daily shipments from local, regional, national, and international farmers. The produce distributor conducted 176 markets during a 60-week period, for a period of approximately 5 months per site. The markets averaged 37 shoppers and $306 in sales; produce was sold at 15% to 25% below retail (local supermarket) prices. Before each market, Brown University staff provided the site contacts at the sponsoring community organizations with promotional flyers and brochures in English and Spanish. The organization’s staff posted these inside the building, distributed them directly to parents, or sent them home with children. The research staff also posted flyers advertising the markets at central neighborhood locations. At each market, the research staff collected and compiled sales and participation data.

### Cohort study

Participant recruitment was conducted by the research staff during a 6-month period during the first Fresh to You markets at each site. A new site started every 6 weeks. For a family to be eligible, adults had to be 18 years or older, had to be parents or legal guardians of a child aged 3 to 13, had to live with the child at least 75% of the time, and had to be knowledgeable about the child’s diet. Eligible parents completed an interviewer-administered baseline survey at the first market they attended and a follow-up survey approximately 5 months later. Parents received incentives of $10 for completing the baseline survey and $20 for completing the follow-up survey.

Survey questions were translated into Spanish; cognitive assessment testing (15) was conducted with the target population, and measures were adapted on the basis of these findings to ensure that the tools were culturally and linguistically appropriate. Children’s fruit and vegetable intake was reported by parents by using a validated fruit and vegetable food frequency questionnaire (16). On the basis of findings from cognitive assessment testing, we made minor modifications to the questionnaire. We originally had 3 timeframes (breakfast, lunch, and dinner), but parents reported that it was easier for them to report children’s intake accurately when there were 5 timeframes (morning, lunchtime, afternoon, dinner time, and evening after dinner). We therefore added the 2 additional timeframes. Additionally, we changed the measurement for fruits and vegetables from servings to cups to align with the 2010 national dietary guidelines (17). Finally, we included size categories appropriate for young children (<1/2 cup, 1/2 cup to ≤1 cup, 1 cup to≤2 cups, and >2 cups). The 5-month, postintervention survey with parents included questions about what they liked about the markets and how the markets could be improved.

### Focus groups and key informant interviews

Six postintervention focus groups were conducted with a subset of 30 parents who attended the markets. Parents were recruited at participating community organizations by using flyers and posters and by face-to-face contact. Focus groups were conducted according to standard focus group procedures (18) in both English and Spanish by trained Brown University research staff. Each focus group lasted approximately 2 hours; healthful refreshments were provided, and participants were compensated with $25 gift cards. We also conducted individual interviews with community organization representatives. The purpose of these focus groups and interviews was to gather feedback about the markets and recommendations for improvement. All focus groups and interviews were audio-recorded. Audiotapes were transcribed and subjected to several stages of analytic coding by Brown University research staff by using ethnographic methods (19).

### Statistical analysis

We tabulated descriptive statistics, frequencies, and cross frequencies for participant demographic characteristics and process evaluation questions. For children’s fruit and vegetable intake, baseline and 5-month follow-up data were compared by using paired *t* tests. We analyzed the data looking at fruits alone (without juice), vegetables alone (without potatoes), and fruits and vegetables together. All analyses were completed by using SAS version 9 (SAS Institute, Inc).

## Results

Of the 480 parent–child pairs recruited at baseline, 79% (n = 378) were retained at 5-month follow-up. Most of the cohort parents were female (91.5%), Hispanic (59.2%), born outside the United States (51.5%), and younger than 40 (67.7%). The annual household income of 70.7% of parent participants was less than $30,000. Educational levels were mixed: 25.1% of participants had less than a high school degree, 30.5% were high school graduates, 21.9% had some college, and 22.6% had college degrees or more (Table 2).

**Table 2:** Demographic Characteristics of Parent–Child Pairs (N = 480) Participating in Fresh to You Market Evaluation Study in Low-Income Neighborhoods, Rhode Island, 2010–2011

Characteristic	%^a^
**Female**	91.5
**Born outside the United States**	51.5
**Hispanic ethnicity **	59.2
**Other race/ethnicity**	
White	33.9
Black	18.6
Native American	2.6
Asian	2.8
Mixed race or other	42.1
**Marital status**	
Single	41.3
Married	39.2
Other	19.6
**Parent age, y**	
18–29	24.4
30–39	43.3
≥40	32.3
**Child age, y**	
3–5	20.2
6–9	48.0
10–13	31.8
**Employment status**	
Full-time	31.1
Part-time	15.1
Unemployed	52.8
**Education**	
Less than high school degree	25.1
High school degree	30.5
Some college	21.9
College or more	22.6
**Annual household income,^a^ $**	
<20,000	51.2
20,000–29,999	19.5
30,000–39,999	11.5
≥40,000	17.8
**Supplemental Nutritional Assistance Program participant**	49.4
**Special Supplemental Nutrition Program for Women, Infants, and Children participant**	30.4

a Some categories do not add to 100%, either because data did not include missing or “don’t know” responses or because of rounding.

Shoppers paid by cash (57.4%), SNAP-EBT (electronic benefits transfer) card (17.4%), debit or credit card (25.1%), or check (1.1%). The top-selling fruits and vegetables were bananas, navel oranges, grapes, tomatoes, cucumbers, kiwi fruit, pears, apples, red and green peppers, and grapefruit. From baseline to 5-month follow-up, there was a significant increase in children’s fruit and vegetable consumption. Consumption increased for fruit without juice (0.20 cups, *t* = 3.00, *P* = .003), vegetables without potatoes (0.28 cups, *t* = 3.61, *P* = .001), and fruits and vegetables combined (0.48 cups, (*t* = 4.16, *P* < .001). When asked what they liked about the markets, 58% said the quality of the fruits and vegetables, 33% said convenience, 27% said variety, and 22% said affordability. Finally, 65% of participants reported that they would be very likely to shop at a mobile Fresh to You market that came to their neighborhood, and 66% said having locally grown produce at the markets was very important to them.

### Qualitative data

**Focus groups.** Thirty parents participated in the 6 post-intervention focus groups. Most were women (96.7%) aged 40 to 49 years (53.3%) and Hispanic (80%) (33.3% Dominican, 16.7% Guatemalan, 13.3% Colombian, 10% Mexican, and 6.7% Puerto Rican). One third (33.3%) reported their race as mixed, 26.7% as white, 13.3% as black, and the rest as unknown. Educational attainment was mixed: 23.3% had less than a high school education, 26.7% had a high school degree, 40% had some college or technical school, and 6.7% had a college degree or more.

Focus group participants reported that they liked the Fresh to You markets because they increased their access to affordable, high-quality fruits and vegetables and enticed their children to eat more fruits and vegetables. They remarked that the produce was much fresher, better quality, and lasted longer without spoiling than the fruits and vegetables at local bodegas and discount stores. They also liked being able to purchase small quantities of produce at affordable prices instead of having to buy it in bulk at the discount food stores. However, some participants told us that while Fresh to You produce prices were lower than supermarket prices, they were higher than discount food store prices. For participants, price was more important than quality. Suggestions for improvement were better advertising of the markets, selling more ethnic produce, providing educational activities, having lists showing comparison of Fresh to You versus supermarket prices, and offering the markets at rotating times and locations. 

**Community organization staff interviews.** Eight interviews were conducted with staff members from each of the community organizations hosting the markets: 2 at the job training center, 2 at the health center, and 1 at each of the 4 school sites. All interviewees were women. No other demographic data were collected. Overall, staff members were positive about the markets and how they fit into their organizations’ missions. They reported that Fresh to You met their expectations for low prices, high quality, and convenience of the markets, being located at their community organizations. Problems cited were that site staff did not have time to promote the markets, the need for even lower prices, and some parents’ difficulties attending the markets because of childcare or work responsibilities.

Their suggestions for improvement were similar to those made by participants. Additional suggestions were offering discount cards to frequent shoppers, opening markets to shoppers outside the site to increase sales, involving local high school students as market staff, and having large quantities of popular produce, so markets did not run out of these products. The site staff also suggested rotating times and locations of markets to make them more convenient for people unable to attend because of work or child care responsibilities. The staff reported positive feedback from their clients who attended the markets: participants liked the quality of the produce and the helpful staff. The staff wanted the Fresh to You markets to continue because they felt they motivated their clients to improve their diets.

## Discussion

Bringing markets with affordable, high-quality produce to convenient locations in ethnically diverse, low-income neighborhoods was an effective strategy for increasing fruit and vegetable intake among children aged 3 to 13 years whose parents shopped at the markets. Results from a follow-up survey and focus groups indicated that most participants appreciated the markets, found them convenient, and valued the high quality and variety of produce sold, in contrast to the poor quality and limited variety available in neighborhood stores. Participants felt that the markets helped improve their families’ diets. However, although fruit and vegetable prices at Fresh to You markets were lower than supermarket prices, affordability remained an issue for the families in our study.

One potential strategy for lowering prices is financial incentives. The 2014 Farm Bill (20) created the Food Insecurity and Nutrition Incentive Program (FINIP), which provides grants to incentivize the purchase of fruits and vegetables by SNAP recipients. Fresh to You, which is now a nonprofit dissemination program under the Rhode Island Public Health Institute, was recently awarded a FINIP pilot grant that will double SNAP recipients’ benefits for the purchase of fruits and vegetables. Another potential strategy for lowering market costs and increasing Fresh to You’s potential to be self-sustaining would be to use college students as volunteer market staff, an approach being explored with Brown University’s Swearer Center for Public Service. Fresh Moves, a Chicago mobile market, used student volunteers, and participants reported that interacting with local students fostered feelings of community (21).

Switching to a mobile market could also reduce costs. In our study, Fresh to You required hours of staff time to haul produce in and out of the truck and to set up and break down markets. In contrast, a mobile market could drive through neighborhoods, stopping at specific sites at predetermined times to sell fruits and vegetables directly from the truck. Fresh to You study participants expressed interest in this model, which would also address their recommendations for rotating market times and locations to accommodate shoppers’ schedules. Under the Fresh to You program, a car trailer was recently retrofitted to serve as a mobile market; this mobile market approach is being studied in 2 ongoing, randomized controlled trials.

Mobile markets are being used across the United States as a strategy for increasing access to healthful food in food deserts (22). Although statistical models demonstrate their potential for increasing access to fruits and vegetables (23,24), research is lacking on resulting improvements in diet. One cross-sectional study at 2 senior-housing complexes demonstrated the effectiveness of a van selling fruits and vegetables at below-retail prices, which increased fruit and vegetable intake by 0.37 servings per day (25), which is equivalent to 0.185 cups — less than the approximate 0.5-cup increase achieved in this study. However, more rigorous, longitudinal studies are needed to evaluate both effectiveness and financial sustainability. We are completing such an evaluation in an NIH-funded, cluster-randomized controlled trial at 15 public housing developments.

Although a detailed business plan is beyond the scope of this article, sustainability for Fresh to You is possible through a “Robin Hood” model, whereby the profit from higher sales (and potentially higher prices) at Fresh to You markets at worksites, colleges, and in high-income neighborhoods could help subsidize the cost of bringing markets to low-income neighborhoods. We successfully brought Fresh to You markets to 14 worksites as part of a randomized trial and have also brought these markets to a park near Brown University and the Rhode Island School of Design where sales have been high. Other colleges and universities have also expressed interest in having Fresh to You markets on their campuses. In addition, we are in the process of creating an online ordering website, which could also increase sales. With these proposed changes, Fresh to You could become a self-sustaining intervention and a model that could be disseminated to other cities and states. Other funding mechanisms such as the Healthy Food Financing Initiative (26) could help support Fresh to You as a mobile retail outlet to expand access to healthy, fresh foods in low-income, underserved communities.

Fresh to You participants reported that they would like to have more local produce at the markets. Thus, creating mutually beneficial partnerships between Fresh to You and local farmers, food hubs, and urban market gardeners is another area we are exploring. Fresh to You could provide a new and expanded distribution system for local produce, especially desirable ethnic produce grown by market gardeners, community gardeners who sell some of what they grow. However, low-income residents need access to affordable fresh fruits and vegetables year-round, not just during the limited Rhode Island growing season. Fresh to You markets are able to address this need because they provide local and nonlocal fruits and vegetables year-round.

This study did not include funding for an educational component, but both parents and site contact indicated that such a component would be helpful. Other researchers have suggested that education should accompany produce markets to affect consumption significantly (27). In 2 ongoing studies, we are evaluating the inclusion of educational programming along with Fresh to You markets. However, the financial cost of educational programs may not be feasible in the long term unless such programs are provided through agencies already funded to do this work, such as the Cooperative Extension Service. Less costly ways to provide education could include point-of-purchase signage, bar code scans that automatically send educational material links to customers via text or email, or use of student volunteers to provide educational activities at the markets. These ideas should be explored in future studies.

Strengths of this study include the combination of qualitative and quantitative data, extensive process evaluation, use of validated tools, cognitive assessment testing of surveys with the target population, and conduct of the intervention and evaluation in Spanish and English. A limitation of this study was the lack of a comparison group. The design could be improved by including a matched comparison community in which parent–child pairs are interviewed but do not receive a market intervention. In addition, randomized trials of fruit and vegetable market interventions such as those we are currently conducting will further inform the field. Another potential study limitation is seasonality; however, because sites were enrolled on a rolling basis every 6 weeks, markets were held in each season of the year with no significant differences noted in our analyses based on season. Findings are also limited by the use of self-reported measures and by our inability to determine whether the increased fruit and vegetable intake replaced existing calories from unhealthful food or represented added calories. Future research should examine this issue.

Fresh to You shows promise as a year-round solution for increasing access to and consumption of fruits and vegetables for low-income, racially/ethnically diverse children. Suggested refinements discussed above should be made, and more rigorous studies should be conducted to evaluate the efficacy of Fresh to You at improving diet among different demographic groups and whether increases in fruit and vegetable consumption replace calories from unhealthful food. Translational research is also needed to explore and evaluate different models for implementation, sustainability, and dissemination.
