# Erratum, Vol. 12, October 15 Release

**DOI:** 10.5888/pcd12.140583e

**Published:** 2015-11-05

In the article “Effectiveness of Fresh to You, a Discount Fresh Fruit and Vegetable Market in Low-Income Neighborhoods, on Children’s Fruit and Vegetable Consumption, Rhode Island, 2010–2011,” we inadvertently listed an author affiliation for Sara Gorham incorrectly. Ms. Gorham is affiliated with the Institute for Community Health Promotion, Brown University School of Public Health, Providence, Rhode Island. The changes were made to our website on October 16, 2015, and appear online at http://www.cdc.gov/pcd/issues/2015/14_0583.htm. We regret any inconvenience or confusion this error may have caused.

